# Metabolic Engineering of Histidine Kinases in *Clostridium beijerinckii* for Enhanced Butanol Production

**DOI:** 10.3389/fbioe.2020.00214

**Published:** 2020-03-20

**Authors:** Xin Xin, Chi Cheng, Guangqing Du, Lijie Chen, Chuang Xue

**Affiliations:** School of Bioengineering, Dalian University of Technology, Dalian, China

**Keywords:** *Clostridium beijerinckii*, CRISPR-Cas9n, histidine kinases, butanol, sporulation

## Abstract

*Clostridium beijerinckii*, a promising industrial microorganism for butanol production, suffers from low butanol titer and lack of high-efficiency genetical engineering toolkit. A few histidine kinases (HKs) responsible for Spo0A phosphorylation have been demonstrated as functionally important components in regulating butanol biosynthesis in solventogenic clostridia such as *C. acetobutylicum*, but no study about HKs has been conducted in *C. beijerinckii*. In this study, six annotated but uncharacterized candidate HK genes sharing partial homologies (no less than 30%) with those in *C. acetobutylicum* were selected based on sequence alignment. The encoding region of these HK genes were deleted with CRISPR-Cas9n-based genome editing technology. The deletion of *cbei2073* and *cbei4484* resulted in significant change in butanol biosynthesis, with butanol production increased by 40.8 and 17.3% (13.8 g/L and 11.5 g/L vs. 9.8 g/L), respectively, compared to the wild-type. Faster butanol production rates were observed, with butanol productivity greatly increased by 40.0 and 20.0%, respectively, indicating these two HKs are important in regulating cellular metabolism in *C. beijerinckii*. In addition, the sporulation frequencies of two HKs inactivated strains decreased by 96.9 and 77.4%, respectively. The other four HK-deletion (including *cbei2087, cbei2435, cbei4925*, and *cbei1553*) mutant strains showed few phenotypic changes compared with the wild-type. This study demonstrated the role of HKs on sporulation and solventogenesis in *C. beijerinckii*, and provided a novel engineering strategy of HKs for improving metabolite production. The hyper-butanol-producing strains generated in this study have great potentials in industrial biobutanol production.

## Introduction

During the past decades, with concerns about diminishing petroleum reserves and fluctuations in oil prices, renewable biofuels have gained intensive attentions. As a substitute for gasoline, butanol has promising physical features such as high energy density, low volatility, and less corrosivity, but clostridia-based biobutanol production is economically unfavorable due to low product titer and productivity (Xue et al., 2013; Yang et al., 2018). *Clostridium beijerinckii* is an important industrial microorganism which produces butanol, but is notoriously difficult to metabolically engineer and hard to break through the limitation of low product concentration (Xue et al., 2017a). Since CRISPR-Cas9 has been explored as a powerful and effective tool for genome editing of lots of organisms including *C. beijerinckii* (Li et al., 2016; Wang et al., 2016a), we expected to focus on editing the genome of *C. beijerinckii* based on this system.

*Clostridium beijerinckii* is an organism historically used for ABE (acetone, butanol, ethanol) fermentation. Batch ABE fermentation is characterized by two distinctive phases, acidogenesis and solventogenesis (Herman et al., 2017). During exponential growth, short-chain fatty acids, including acetic acid and butyric acid, are produced and accumulated in the system, which causes a drop in the culture pH. As the culture reaches a low enough pH, the formed acids can be re-assimilated and the culture pH rises. At the same time, solvent production is initiated (Li et al., 2011). The metabolic transition from acidogenesis to solventogenesis is consistent with the initiation of the complex sporulation process. During sporulation, a starch-like carbohydrate called granulose accumulates in the form of a swollen, bright-phase *Clostridium* bacterium, in which the endospore begins to develop. Further morphological development produces free-spores, heat- and chemical-resistant cell types that do not contribute to solvent production (Al-Hinai et al., 2015; Herman et al., 2017).

Histidine kinases (HKs) are involved in perceiving and transducing environmental signals to trigger multiple cellular responses, such as cell division (Jacobs et al., 1999), nitrogen metabolism (Loomis et al., 1997), antibiotic resistance (Wilke et al., 2015) and sporulation (Xue and Cheng, 2019). Orphan HKs are HKs lacking an adjoining response regulator, as is typical for most HKs in prokaryotes (Steiner et al., 2011). Several orphan HKs, regulating spore development in *C. perfringens* (Hiscox et al., 2013), *C. difficile* (Underwood et al., 2009), *C. botulinum* (Wörner et al., 2006) and *C. thermocellum* (Mearls and Lynd, 2014), etc., have been identified. Especially, orphan HKs directly phosphorylate Spo0A, which is the master regulator of both sporulation and solventogenesis in *C. acetobutylicum* (Steiner et al., 2011). Spo0A directly controls the genes with the presence of one or more “0A boxes” (TGNCGAA) in their 5′ regulatory regions (Wilkinson et al., 1995). The “0A boxes” are present in the upstream regions of many genes involving sporulation such as *sigE* and *spoIIE*, and central metabolism such as *adc*, *sol* operon, *bdhA* and *bdhB* (Wilkinson et al., 1995; Kolek et al., 2017). To regulate the transcription of these genes, the phosphorylated Spo0A will bind to the “0A boxes” to function. In *C. acetobutylicum*, there are two pathways for Spo0A activation: one depends on a HK Cac0323, and the other involves two HKs Cac0903 and Cac3319, respectively (Steiner et al., 2011). Individual mutants of the three HKs inactivation showed that the sporulation frequency was decreased by 95–99% compared to the wild-type strain (Steiner et al., 2011), indicating these HKs have similar characteristics and are essential for spore development. More importantly, inactivation of Cac3319 in *C. acetobutylicum* was found to give positive phenotypic changes, including increased butanol tolerance and production (Xu et al., 2015). However, no relevant study has been performed in *C. beijerinckii*, another important butanol-producing strain that could more preferably utilize lignocellulosic hydrolysate (Xiao et al., 2012). There are 85 HKs in the genome of *C. beijerinckii* and only one of them has been investigated to explore its regulatory function. The three-component system consists of HK LytS and response regulator YesN, which directly regulates the transcription of *xylFGH* genes and is responsible for xylose transportation (Sun et al., 2015). However, whether orphan HKs could regulate sporulation and solventogenesis in *C. beijerinckii*, as they did in *C. acetobutylicum*, was still unknown. The genome sequence data indicated that clostridia largely shared the key HK genes for phosphorylation of Spo0A (Al-Hinai et al., 2015), and thus we hypothesized that analogous HKs existed and were functional in *C. beijerinckii*.

In this study, we reported the identification and verification of orphan HKs in *C. beijerinckii* for their regulatory function in solventogenesis and sporulation. Six HK candidates were identified by sequence alignment with *cac3319*, *cac0323*, and *cac0903* in *C. acetobutylicum*. Using CRISPR-Cas9n system, we successfully deleted these HK genes, and fermentation analysis showed that two of them were important for regulating butanol biosynthesis and spore development. This study demonstrated the role of HKs on sporulation and solventogenesis in *C. beijerinckii* for the first time. The HK-engineered strains can be used as robust workhorses for enhanced butanol production, and also can be further engineered for production of valuable metabolites.

## Materials and Methods

### Bacterial Strains, Culture Conditions, and Plasmids

All bacterial strains and plasmids used in this study were listed in Supplementary Table S1. The *Escherichia coli* DH5α was used for plasmid cloning. The *E. coli* transformants were grown aerobically at 37°C in Luria-Bertani (LB) medium or on solid LB agar (2% w/v) plate supplemented with ampicillin (100 μg/mL) when necessary. *C. beijerinckii* CC101 was an adaptive mutant of *C. beijerinckii* NCIMB 8052 (ATCC 51743) from ST Yang’s lab (Lu et al., 2013), and its mutant strains were grown anaerobically at 37°C in Clostridium Growth Medium (CGM) (Li et al., 2016) supplemented with erythromycin (40 μg/mL) as necessary.

### Reagents and Enzymes

All restriction enzymes used in this study were purchased from New England Biolabs (Beverly, MA, United States). The DNA polymerase KOD FX (Toyobo, Osaka, Japan) was used for DNA amplification and colony extension PCR to screen the positive transformants. Recombinant plasmids were assembled through the ClonExpress One Step Cloing Kit (Vazyme Biotech, Nanjing, China).

### Plasmid Construction

All the DNA oligonucleotides used in this study were synthesized in Sangon Biotech Co., Ltd. (Shanghai, China) and listed in Supplementary Table S2. The schematic diagram of the plasmid construction and gene-editing process was shown in Supplementary Figure S1. CRISPR-based genome editing plasmid, pNICKclos 2.0-*xylR* (Li et al., 2016), was used as the vector to delete HK genes. As the selected target region (N20-NGG) was crucial for sgRNA targeting, alignment research (NCBI BLAST) was performed to ensure that the 23-bp target sequence had no sequence similarity elsewhere in the genome and thus could avoid multiple off-target candidates. The sgRNA-encoding region targeting the *cbei2073* was placed following the Pj23119 synthetic promoter through the steps described below. The primers pNICKclos-2073-1-1/pNICKclos-2073-3 were used to generate a Pj23119-sgRNA-2073-1 cassette from pNICKclos 2.0-*xylR* and then the cassette was used as the template to PCR amplify the Pj23119-sgRNA-2073-2 cassette with primers pNICKclos-2073-1-2/pNICKclos-2073-3. The 1.2-kb upstream and 1.2-kb downstream homology arms (HAs) of the selected target region (N20-NGG) in *cbei2073* were amplified from the genome of *C. beijerinckii* CC101 using primers pairs pNICKclos-2073-2/pNICKclos-2073-5 and pNICKclos-2073-4/pNICKclos-2073-6, respectively. Then the Pj23119-sgRNA-2073-2 cassette and the two HAs were joined by overlap extension PCR using primers pNICKclos-2073-1-2/pNICKclos-2073-6, generating a fragment in which the two HAs were separated by a *Pst*I restriction site. Finally, the fragment was fused with the *Spe*I/*Xho*I linearized pNICKclos 2.0-*xylR* plasmid through the One Step Cloning Kit, yielding pNICKclos 2.0-*cbei2073*. All the other plasmids (pNICKclos 2.0-*cbei2087*, pNICKclos 2.0-*cbei2435*, pNICKclos 2.0-*cbei1553*, pNICKclos 2.0-*cbei4925*, and pNICKclos 2.0-*cbei4484*) used for gene deletion based on CRISPR-Cas9 nickase in *C. beijerinckii* CC101 were also derived from pNICKclos 2.0-*xylR* using similar methods as described above.

### Transformation of *C. beijerinckii*

Plasmids for genome editing were transformed into *C. beijerinckii* via electroporation (Mermelstein et al., 1992) according to the following protocol. To prepare electrocompetent cells, 2 mL stock culture in 20% glycerol was inoculated into 100 mL CGM medium until the optical density at 600 nm (OD_600_) reached 0.4–0.6. All of the following steps were done at low temperatures (4°C) and the buffers used were pre-cooled. The culture was centrifuged at 4500 r/min for 10 min and then the cells were resuspended in 30 mL ETM buffer (270 mM sucrose, 4.4 mM NaH_2_PO_4_, 0.6 mM Na_2_HPO_4_, 10 mM MgCl_2_, pH 7.4) for 10 min. The resuspended cells were centrifuged and resuspended in 2.5 ml ET buffer (270 mM sucrose, 4.4 mM NaH_2_PO_4_, 0.6 mM Na_2_HPO_4_, pH 7.4). The mixture of 190 μL competent cells and 10 μL plasmid DNA (1–2 μg) was transferred to an electroporation cuvette with a 0.2 cm gap width. Electroporation was performed using a Bio-Rad Micropulser (Bio-Rad Laboratories, Hercules, CA, United States) at 1.8 kV. After electroporation, samples were mixed with 400 μL CGM immediately and transferred to another 400 μL CGM in a 2-mL tube. After incubation for 4–6 h at 37°C, 200 μL recovered cultures were plated on CGM agar containing 40 μg/mL erythromycin and grown anaerobically at 37°C for 48 h. Transformant colonies were picked from the agar plates and subjected to PCR-based verification.

### Mutant Screening

Individual transformant colonies were all picked and analyzed using colony-PCR after electroporation. Then the PCR productions were digested by restriction endonuclease and sequenced to confirm the positive transformant colonies. The primers used were selected from sequence located about 100 bp up- and down-stream of the HAs on the genome named *cbei2073*-For/*cbei2073*-Rev, etc. The restriction site (*Pst*I) between the two HAs in the editing template was confirmed by cleavage of the fragment amplified by colony extension PCR with *Pst*I restriction enzyme. Parent strain *C. beijerinckii* CC101 genome was used as a negative control.

### Plasmid Curing

To remove the pNICKclos 2.0 plasmids from mutants, the positive transformants were cultured in 5 mL of CGM liquid medium (M1) without antibiotic pressure at first. After growing for 12 h, 50 μL of M1 was inoculated into 5 mL CGM medium without antibiotic (M2). After another 12 h, the OD_600_ of M2 increased to ∼0.8. Then 100 μL of the M2 broth was spread on a CGM medium ager plate without antibiotic. After 12 h, the individual colonies were spread onto CGM ager with erythromycin (50 μg/mL) and CGM ager without antibiotic. The cells that grew on the later but could not grow on the former were considered to have lost the plasmid.

### Batch Fermentation

Batch fermentations with various engineered *C. beijerinckii* strains were performed anaerobically in P2 medium in a 3-L bioreactor with 1.5-L working volume. The P2 medium was prepared as previously described (Xue et al., 2016), which contained glucose ∼90 g/L; yeast extract 1 g/L; CH_3_COONH_4_ 2.2 g/L; K_2_HPO_4_ 0.5 g/L; KH_2_PO_4_ 0.5 g/L; MgSO_4_⋅7H_2_O 0.2 g/L; MnSO_4_⋅H_2_O 0.01 g/L; FeSO_4_⋅7H_2_O 0.01 g/L; NaCl 0.01 g/L; para-amino-benzoic acid 1 mg/L; thiamin 1 mg/L; and biotin 1 mg/L. The *C. beijerinckii* strains were first incubated anaerobically in 100 mL of CGM at 37°C until OD_600_ reached ∼1.0 and then the seed culture was transferred into the bioreactor containing 1.4 L P2 medium. All the media used in this study were sterilized at 121°C and 15 psig for 15 min and purged with N_2_ for 20 min through a sterile 0.2 μm filter to remove dissolved oxygen.

### Sporulation Frequency Assay

The strains were cultured in liquid CGM medium for 5 days. Then the cell cultures were treated in 80°C for 10 min, after which 100 μL of them were plated on CGM agar directly or following diluting for 10 times. The sporulation frequency of strains was characterized by the numbers of heat-resistant colonies.

### Analytical Methods

The fermentation samples were periodically taken from the bioreactor and the cell growth was analyzed by measuring OD_600_ using a spectrophotometer (Thermo Spectronic, United States). Samples for glucose and product analyses were pelleted by centrifugation at 8000 × *g* for no less than 5 min. The solvents of the fermentation broth (acetone, butanol, and ethanol) were analyzed using a gas chromatograph (Agilent 6890A GC, United States) as previously described (Xue et al., 2017b; Yang et al., 2019). Glucose, acetate and butyrate were analyzed by HPLC (Waters 1525, United States) equipped with an Aminex HPX-87H column (300 mm × 7.8 mm) maintained at 50°C. Dilute H_2_SO_4_ (10 mM, 0.5 mL/min) was used as the eluent (Xiao et al., 2019).

## Results

### Screening of Target Genes and Construction of Engineered Strains

**FIGURE 1:**
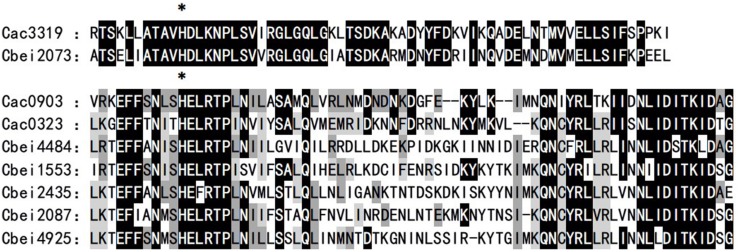
Comparison of HK phosphodonor active site domains. The DHp/HisKA domains of several putative orphan HKs in *C. beijerinckii* were compared to known orphan kinases Cac3319, Cac0903, and Cac0323 (*C. acetobutylicum*), respectively. Identical residues conserved with Cac3319 or Cac0903 and Cac0323 are shaded black, identical residues conserved with Cac0903 are shaded dark gray, and identical residues conserved with Cac0323 are shaded light gray. The phosphorylated histidine residue is denoted by an asterisk.

To explore the uncharacterized HKs in *C. beijerinckii*, sequence alignments between the nucleotide sequence of three orphan HK genes in *C. acetobutylicum* (*cac3319*, *cac0323*, and *cac0903*) and the whole genome of *C. beijerinckii* were performed. Six candidates, including *cbei2073*, *cbei2087*, *cbei4925*, *cbei2435*, *cbei1553*, and *cbei4484*, were identified. Among them, *cbei2073* showed 46% homology with *cac3319*, and the others showed 31–36% homology with *cac0323* or *cac0903* gene. By further amino acid sequence alignment of HisKA domain, Cbei2073 showed 78% sequence similarity with Cac3319 and the others showed no less than 70% with Cac0903 or Cac0323. Also, Cbei2073 was special among the six in that its phosphorylation sites and their adjacent regions were highly conserved and similar to Cac3319, which has been proved to be the most functional HK in regulating butanol biosynthesis in *C. acetobutylicum* (Figure 1; Xu et al., 2015). Based on these analyses, these six HKs were finally selected as candidate homologous proteins.

After selecting these targeting genes, recombinant plasmids (pNICKclos 2.0-*cbei2073*, pNICKclos 2.0-*cbei2087*, pNICKclos 2.0-*cbei2435*, pNICKclos 2.0-*cbei1553*, pNICKclos 2.0-*cbei4925*, and pNICKclos 2.0-*cbei4484*) were first constructed to delete the corresponding gene, respectively. Then they were separately transferred into *C. beijerinckii* via electroporation. A low number of transformants could been identified after electroporation, but the verification results showed that the accuracy rates (number of correctly edited transformants/total number of transformants screened) were no less than 60% (Supplementary Table S3).

### Impact of HK Genes on ABE Fermentation

**FIGURE 2:**
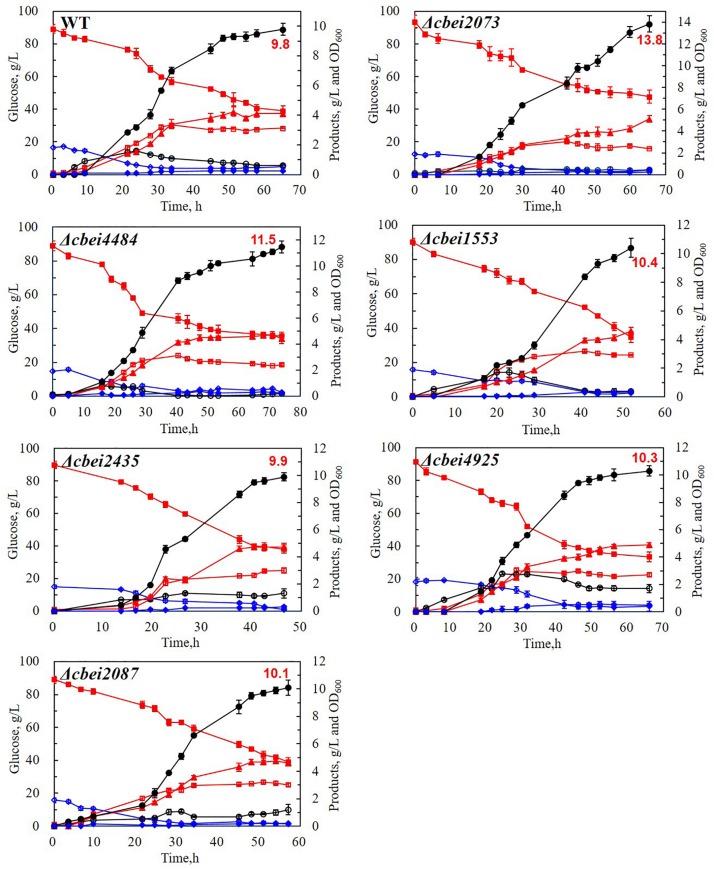
Fermentation results of the wild-type (WT) and mutant strains in batch fermentation. Symbols: glucose (filled squares), OD_600_ (open squares), butanol (filled circles), acetone (filled triangles), ethanol (filled diamonds); butyrate (open circles), acetate (open diamonds).

To investigate the effect of HKs on solventogenesis and cell growth, batch fermentation performances of wild-type *C. beijerinckii* and HK-inactivated strains were compared (Figure 2 and Table 1). Cbei2073 inactivated strain showed enhanced butanol production with 40.8 and 40.0% increases in butanol titer and butanol productivity, respectively, compared to wild-type *C. beijerinckii*. In addition, increases in ethanol and acetone were also observed in Cbei2073 inactivated strain. According to the fermentation kinetics study, it could be concluded that Cbei2073 played an important role in regulating solvent production, which was similar to HK Cac3319 of *C. acetobutylicum*. Cbei4484 inactivated strain showed enhanced butanol production with 17.3 and 20.0% increases in butanol titer and butanol productivity, respectively. Fermentation of Cbei1553 mutant strain showed that this HK had minor effects on cell growth and solvent production, but its inactivation resulted in 40.0% increase in butanol productivity. The ABE titers of Cbei2435, Cbei2087, and Cbei4925 inactivated strains were similar to that of the wild strain. However, Cbei2435 and Cbei2087 mutant strains showed slight increases in butyrate titer and significant increases in butanol productivity.

**TABLE 1:** Fermentation parameters of wild-type and mutant strains in batch fermentation.

Strains	OD_600 (max)_	Glucose consumption (g/L)	Acids (g/L)		Solvents (g/L)				Yield (g/g)		Productivity (g/L/h)	
						
			Acetic acid	Butyric acid	Acetone	Butanol	Ethanol	ABE	Butanol	ABE	Butanol	ABE
CC101	3.1 ± 0.1	50.1 ± 3.1	0.5 ± 0.1	0.6 ± 0.1	4.1 ± 0.2	9.8 ± 0.4	0.2 ± 0.0	14.1 ± 0.7	0.20 ± 0.02	0.28 ± 0.03	0.15 ± 0.02	0.22 ± 0.03
Δ*cbei2073*	3.0 ± 0.1	58.4 ± 4.2	0.2 ± 0.0	0.4 ± 0.0	5.1 ± 0.3	13.8 ± 0.8	0.4 ± 0.1	19.4 ± 0.7	0.24 ± 0.02	0.33 ± 0.02	0.21 ± 0.02	0.30 ± 0.03
Δ*cbei4484*	3.1 ± 0.2	53.5 ± 2.7	0.3 ± 0.0	0.2 ± 0.0	4.4 ± 0.4	11.5 ± 0.5	0.3 ± 0.0	16.1 ± 0.5	0.21 ± 0.01	0.30 ± 0.01	0.18 ± 0.01	0.25 ± 0.02
Δ*cbei2087*	3.2 ± 0.1	49.9 ± 3.3	0.2 ± 0.0	1.2 ± 0.4	4.6 ± 0.1	10.1 ± 0.6	0.2 ± 0.0	14.9 ± 0.6	0.20 ± 0.00	0.30 ± 0.00	0.18 ± 0.00	0.26 ± 0.00
Δ*cbei2435*	3.0 ± 0.2	51.7 ± 4.1	0.2 ± 0.0	1.3 ± 0.3	4.8 ± 0.3	9.9 ± 0.3	0.3 ± 0.1	15.0 ± 0.5	0.19 ± 0.01	0.29 ± 0.02	0.21 ± 0.02	0.32 ± 0.03
Δ*cbei1553*	3.2 ± 0.1	55.3 ± 4.4	0.3 ± 0.0	0.4 ± 0.0	4.6 ± 0.3	10.4 ± 0.7	0.3 ± 0.0	15.3 ± 0.4	0.19 ± 0.01	0.28 ± 0.02	0.20 ± 0.02	0.29 ± 0.02
Δ*cbei4925*	3.0 ± 0.2	57.7 ± 3.8	0.5 ± 0.1	1.7 ± 0.3	4.9 ± 0.1	10.3 ± 0.4	0.4 ± 0.1	15.6 ± 0.9	0.18 ± 0.01	0.27 ± 0.02	0.16 ± 0.00	0.24 ± 0.01

*The results obtained were presented as the mean ± SD for triplicates of cultures.*

### Effect of HKs on Sporulation Frequency

**FIGURE 3:**
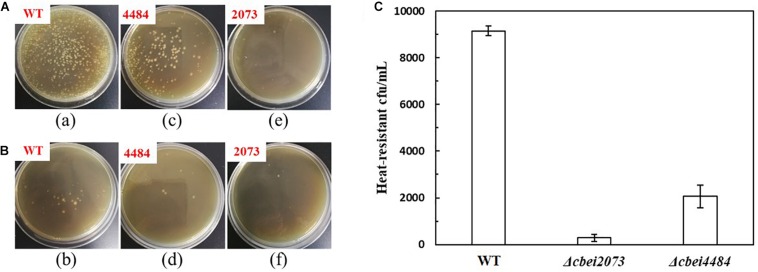
Sporulation frequencies of the wild-type (WT) and mutant strains. The heat-resistant colony-forming units (CFU) were evaluated by treating the cell cultures (5 days) in 80°C for 10 min, after which 100 μL of them were plated on CGM agar directly **(A)** or following diluting for 10 times **(B)**. **(C)** The numbers of heat-resistant colonies.

As spore-forming cells were metabolically inactive, it is hypothesized that the enhanced butanol production was resulted from reduced sporulation frequency caused by inactivation of HK. The heat-resistant colonies of all the six HK mutants and wild-type strain were tested. It was found that the heat-resistant colonies of four HK genes (*cbei2435*, *cbei1553*, *cbei2087*, and *Cbei4925*) deleted strains were in the same level with wild-type strain, which were much more than the other two mutants (Δ*cbei2073* and Δ*cbei4484*). Since *cbei2073* and *cbei4484* had obvious effect on butanol production, we compared the sporulation frequency of wild-type strain, *cbei2073* and *cbei4484* deleted strains by counting the number of heat-resistant colonies following incubation in liquid CGM medium for 5 days. As excepted, significant decreases of sporulation frequency were observed for two HKs inactivated strains, with spore formation reduced by 96.9 and 77.4% of the wild-type strain, respectively (Figure 3). It was indicated that these two HKs might have effects on sporulation and its related genes.

## Discussion

CRISPR-Cas9n based gene editing technology is a method which is not only effective but also widely used in bacteria including *C. acetobutylicum*, *C. beijerinckii*, *Bacillus licheniformis*, etc. (Wang et al., 2015, 2016a,b; Li et al., 2016,^2018^; Zhang et al., 2018). Despite the wide application of this system, it remains reliant on homologous recombination (HR) (Li et al., 2016). In clostridia, HR is notoriously inefficient, which cannot be solved by CRISPR-Cas9 technologies alone (Bruder et al., 2016; Charubin et al., 2018). For this reason, the numbers of individual transformant colonies obtained after the transformation of plasmids into *C. beijerinckii* and the edited cells were both small (Supplementary Table S3). However, the verification results showed that the accuracy rates (60.0–100.0%) were relative higher than those (6.7–100.0%) obtained in a previous study (Li et al., 2016).

Orphan HKs in *Clostridium* species are reported to directly phosphorylate Spo0A, which is the master regulator for initiation of sporulation and solventogenesis (Steiner et al., 2011; Dürre, 2014). To explore the regulatory function of HKs on biobutanol synthesis, the 85 HK genes in *C. beijerinckii* were analyzed and compared with three HKs (Cac3319, Cac0903, and Cac0323) in *C. acetobutylicum* with elucidated functions. Cac3319 inactivated strains could produce 44.4% more butanol (Xu et al., 2015), indicating its vital role in regulating solvent production in *C. acetobutylicum*. In this study, Cac3319 and Cbei2073 were similar based on the sequence alignment results. The deletion of *cbei2073* gene led to significant change in butanol biosynthesis with butanol production increased by 40.8%, indicating that Cbei2073 was also most effective in regulating biobutanol synthesis among the selected six HKs. However, some HKs including Cbei1553, Cbei4925, Cbei2435, and Cbei2087 seemed to have no apparent effect on ABE synthesis, and the growth of all the strains with inactivated HKs was not affected.

In *C. acetobutylicum*, sporulation is closely associated with solventogenesis, and both of them are controlled by Spo0A (Steiner et al., 2011). Cac3319, Cac0903, and Cac0323 are essential for Spo0A activation, which controls the initiation of sporulation and solventogenesis (Steiner et al., 2011; Xu et al., 2015). The “0A box” that can be bound with Spo0A∼P has been proved present in the upstream regions of lots of genes whose expressions are either activated or repressed at the initiation of solventogenesis in both *C. acetobutylicum* and *C. beijerinckii* (Wilkinson et al., 1995). Generally, the sporulating phenotype is considered to be profitable for solvent formation, but solvent production ceases when mature spores form (Kolek et al., 2016). In addition, overexpression of *spo0A* in *C. beijerinckii* NRRL B-598, sharing high genome homology with *C. beijerinckii* NCIMB 8052, led to cessation of production at a low ABE concentration, indicating that Spo0A is a master regulator of solventogenesis in *C. beijerinckii* (Kolek et al., 2017). Furthermore, without spore formation, *C. beijerinckii* NRRL B-598 could produce more butanol in RCM medium, compared with the sporulating phenotype (Branska et al., 2018). Therefore, we speculated that Cbei2073 and Cbei4484 played a role on phosphorylation of Spo0A due to their high similarity in sequence with Cac3319, Cac0903, and Cac0323 and further regulated both butanol synthesis and sporulation frequency. As shown in the results, the inactivation of the two HKs (Cbei2073 and Cbei4484) may lower Spo0A∼P level and then decrease sporulation frequency, finally leading to the improved butanol production. Selective inhibition of sporulation process was beneficial for improving the economics of ABE fermentation since solvent can be produced during a longer time frame (Cheng et al., 2019).

In conclusion, the results of the present study demonstrated that by deleting HK genes, the sporulation frequency might be decreased, with different degrees of improvements in butanol titers, productivities and yields (Table 1). Therefore, this study provided a novel strategy for promoting production of metabolites applicable for a broad of bacteria.

## Data Availability Statement

The raw data supporting the conclusions of this article will be made available by the authors, without undue reservation, to any qualified researcher.

## Author Contributions

CX developed the research scheme. XX performed the experiments and drafted the manuscript. XX, CC, GD, and CX were involved in the data interpretation and result discussion. XX, CC, GD, LC, and CX were involved in the manuscript revision. All authors read and approved the final manuscript.

## Conflict of Interest

The authors declare that the research was conducted in the absence of any commercial or financial relationships that could be construed as a potential conflict of interest.
