# Supplementary material for: Metabolic Engineering of Histidine Kinases in Clostridium beijerinckii for Enhanced Butanol Production
Source: Front Bioeng Biotechnol. 2020 Mar 20;8:214. doi: 10.3389/fbioe.2020.00214 (PMC7098912; doi:10.3389/fbioe.2020.00214)
Supplement: Supplementary file 1 [file Data_Sheet_1.pdf]

Supplementary Material

Supplementary Table 1 Strains and plasmids used in this study

Strains or plasmids	Relevant characteristics	Source or reference
**Bacterial strains**		
C. beijerinckii CC101	NCIMB 8052 (ATCC51743) adaptive mutant	(Lu et al., 2013)
Δcbei2073	*cbei2073*::*Pst*I	This study
Δcbei4484	*cbei4484*::*Pst*I	This study
Δcbei2087	*cbei2087*::*Pst*I	This study
Δcbei2435	*cbei2435*::*Pst*I	This study
Δcbei1553	*cbei1553*::*Pst*I	This study
Δcbei4925	*cbei4925*::*Pst*I	This study
E. coli DH5α	General cloning host strain	Invitrogen
**Plasmids**		
pNICKclos2.0-xylR	Amp, pj23119, sgRNA-*xylR* (8052)	(Li et al., 2016)
pNICKclos2.0-*cbei2073*	Derived from pNICKclos2.0-*xylR*, pj23119, sgRNA-2073 (CC101), homologous arms (*Pst*I was added between the two 1.2-kb arms)	This study
pNICKclos2.0-*cbei4484*	Derived from pNICKclos2.0-*xylR*, pj23119, sgRNA-4484 (CC101), homologous arms (*Pst*I was added between the two 1.2-kb arms)	This study
pNICKclos2.0-*cbei2087*	Derived from pNICKclos2.0-*xylR*, pj23119, sgRNA-2087 (CC101), homologous arms (*Pst*I was added between the two 1.2-kb arms)	This study
pNICKclos2.0-*cbei2435*	Derived from pNICKclos2.0-*xylR*, pj23119, sgRNA-2435 (CC101), homologous arms (*Pst*I was added between the two 1.2-kb arms)	This study
pNICKclos2.0-*cbei1553*	Derived from pNICKclos2.0-*xylR*, pj23119, sgRNA-1553 (CC101), homologous arms (*Pst*I was added between the two 1.2-kb arms)	This study
pNICKclos2.0-*cbei4925*	Derived from pNICKclos2.0-*xylR*, pj23119, sgRNA-4925 (CC101), homologous arms (*Pst*I was added between the two 1.2-kb arms)	This study

Supplementary Table 2 Primers used in this study

Primer name	Sequence (5’-- 3’)
pNICKclos-2073-1-1	CCTAGGTATAATACTAGT**GGCAATGGGGCAGCCAATTT**GTTTTAGAGCTAGAAATAGCA
pNICKclos-2073-1-2	TGAACGCAAGTTTCTAATTTCGGTTACCAATCGATAGAGGAAAGTGTCT
pNICKclos-2073-2	CACCGAGTCGGTGCTTTTTTGCGGCCGCTCCGGAAACTGTCTCAATCTTT
pNICKclos-2073-3	AAAGATTGAGACAGTTTCCGGAGCGGCCGCAAAAAAGCACCGACTCGGTG
pNICKclos-2073-4	ATAATGGAATAGGCACTGCATTCTGCAGTTTATGGGGCAGAACACTATGG
pNICKclos-2073-5	GATATGTAAATTTAAAAAAGTACTGCAGAATAAATAGTATTTTGTATGGT
pNICKclos-2073-6	ATTATTATTTTTATCAATATATTTTGTTAAAAACTCGAGATCGTAGTTTTTATTTTCAG
pNICKclos-4484-1-1	CCTAGGTATAATACTAGT**ATCTCAGATCAGCGCATGCC**GTTTTAGAGCTAGAAATAGCA
pNICKclos-4484-1-2	TGCAGTTGACAGCTAGCTCAGTCCTAGGTATAATACTAGTATCTC
pNICKclos-4484-2	CACCGAGTCGGTGCTTTTTTGCGGCCGCAACGTGAAGAGATAATTGGTCA
pNICKclos-4484-3	TGACCAATTATCTCTTCACGTTGCGGCCGCAAAAAAGCACCGACTCGGTG
pNICKclos-4484-4	AATTTCAGTAAGGAGAAGATAACTGCAGATGGAAATATTAGAATTGAAAA
pNICKclos-4484-5	TTTTCAATTCTAATATTTCCATCTGCAGTTATCTTCTCCTTACTGAAATT
pNICKclos-4484-6	ATTATTATTTTTATCAATATATTTTGTTAAAAACTCGAGGGCTTTAGAGCCTCACCAAC
pNICKclos-2087-1-1	CCTAGGTATAATACTAGT**GTAATAGGGGCAATAGGGTT**GTTTTAGAGCTAGAAATAGCA
pNICKclos-2087-1-2	TGCAGTTGACAGCTAGCTCAGTCCTAGGTATAATACTAGTGTAAT
pNICKclos-2087-2	CACCGAGTCGGTGCTTTTTTGCGGCCGCATTAATGGAAAGGCTTTTGTTT
pNICKclos-2087-3	AAACAAAAGCCTTTCCATTAATGCGGCCGCAAAAAAGCACCGACTCGGTG
pNICKclos-2087-4	AATATTGGATAAAGATAAAAAACTGCAGAAATAAAATTAGTAATGATTAT
pNICKclos-2087-5	ATAATCATTACTAATTTTATTTCTGCAGTTTTTTATCTTTATCCAATATT
pNICKclos-2087-6	ATTATTATTTTTATCAATATATTTTGTTAAAAACTCGAGTCTCCATAAATACTTTTTAC
pNICKclos-2435-1-1	CCTAGGTATAATACTAGT**AAAGCTAATGGCTTGCGATG**GTTTTAGAGCTAGAAATAGCA
pNICKclos-2435-1-2	TGCAGTTGACAGCTAGCTCAGTCCTAGGTATAATACTAGTAAAGC
pNICKclos-2435-2	CACCGAGTCGGTGCTTTTTTGCGGCCGC AGATTTTATTAATAACAAAGAA
pNICKclos-2435-3	TTCTTTGTTATTAATAAAATCTGCGGCCGCAAAAAAGCACCGACTCGGTG
pNICKclos-2435-4	TTTTTGATACAAACGTTGAAGATGTACAATAAAATCGAAAGAATAATGCT
pNICKclos-2435-5	AGCATTATTCTTTCGATTTTATTGTACATCTTCAACGTTTGTATCAAAAA
pNICKclos-2435-6	ATTATTATTTTTATCAATATATTTTGTTAAAAACTCGAGAAATGGAGGTGTTGAATCAA
pNICKclos-1553-1-1	CCTAGGTATAATACTAGT**AGGAAAGTGAGAGACAATTA**GTTTTAGAGCTAGAAATAGCA
pNICKclos-1553-1-2	TGCAGTTGACAGCTAGCTCAGTCCTAGGTATAATACTAGTAGGAA
pNICKclos-1553-2	CACCGAGTCGGTGCTTTTTTGCGGCCGCGATCATCATCCTCAAGTAAAAT
pNICKclos-1553-3	ATTTTACTTGAGGATGATGATCGCGGCCGCAAAAAAGCACCGACTCGGTG
pNICKclos-1553-4	TTTTGAAGAGAGGCTATAAGTACTGCAGAATTATAGATTTTAATTATATT
pNICKclos-1553-5	AATATAATTAAAATCTATAATTCTGCAGTACTTATAGCCTCTCTTCAAAA
pNICKclos-1553-6	ATTATTATTTTTATCAATATATTTTGTTAAAAACTCGAGAAGCCTAATATCTTTTCCAT
pNICKclos-4925-1-1	CCTAGGTATAATACTAGT**ATTAGAGATGCGTCAGAAAG**GTTTTAGAGCTAGAAATAGCA
pNICKclos-4925-1-2	TGCAGTTGACAGCTAGCTCAGTCCTAGGTATAATACTAGTATTAG
pNICKclos-4925-2	CACCGAGTCGGTGCTTTTTTGCGGCCGCTGGATTATAACAGAGTTGTTAA
pNICKclos-4925-3	TTAACAACTCTGTTATAATCCAGCGGCCGCAAAAAAGCACCGACTCGGTG
pNICKclos-4925-4	AGGATGCTGGAGGATTAAGTTACTGCAGTTGAAGGGATAAAAAAGGATTT
pNICKclos-4925-5	AAATCCTTTTTTATCCCTTCAACTGCAGTAACTTAATCCTCCAGCATCCT
pNICKclos-4925-6	ATTATTATTTTTATCAATATATTTTGTTAAAAACTCGAGGGTATGGTGCCGTTCAGCCT
cbei2073-For	CCAGGGGATTCAGGAGTATAA
cbei2073-Rev	CCCATTTGTAATTTTTAAGGC
cbei4484-For	GAGATGAAATAGGGGAATTGGC
cbei4484-Rev	CGGAATCCTTCATCAACAACC
cbei2087-For	CAGGGTCCGACAATTGATATGG
cbei2087-Rev	CTCCCCTAGTGCTCGTCTAAAT
cbei2435-For	TAGCCAAGCCTATATACATAGC
cbei2435-Rev	CCGCATCGCAAGCCATTAGCTTT
cbei1553-For	GATCAAAAGGGAATCCTGC
cbei1553-Rev	CCTGTCTGAGCATCCATCAC

cbei4925-For	CGTAGCCGAAGATGGTATAACG
cbei4925-Rev	GATTTTAATAACACGGCCTCTTG

The bolded bases are the N20 sequences for targeting the genes to be deleted.

Supplementary Table 3 Summary of genome editing results in *C. beijerinckii* BA101

Targets	Deletion size	Insertion sites	Results[a]	Plasmid curing efficiency
cbei2073	23 bp	*Pst*I	2/2	17/22
cbei4484	23 bp	*Pst*I	3/4	18/21
cbei2087	23 bp	*Pst*I	2/3	13/18
cbei2435	23 bp	*Pst*I	3/5	14/18
cbei1553	23 bp	*Pst*I	4/4	17/21
cbei4925	23 bp	*Pst*I	1/1	16/19

[a]Number of correctly edited transformants/total number of transformants screened

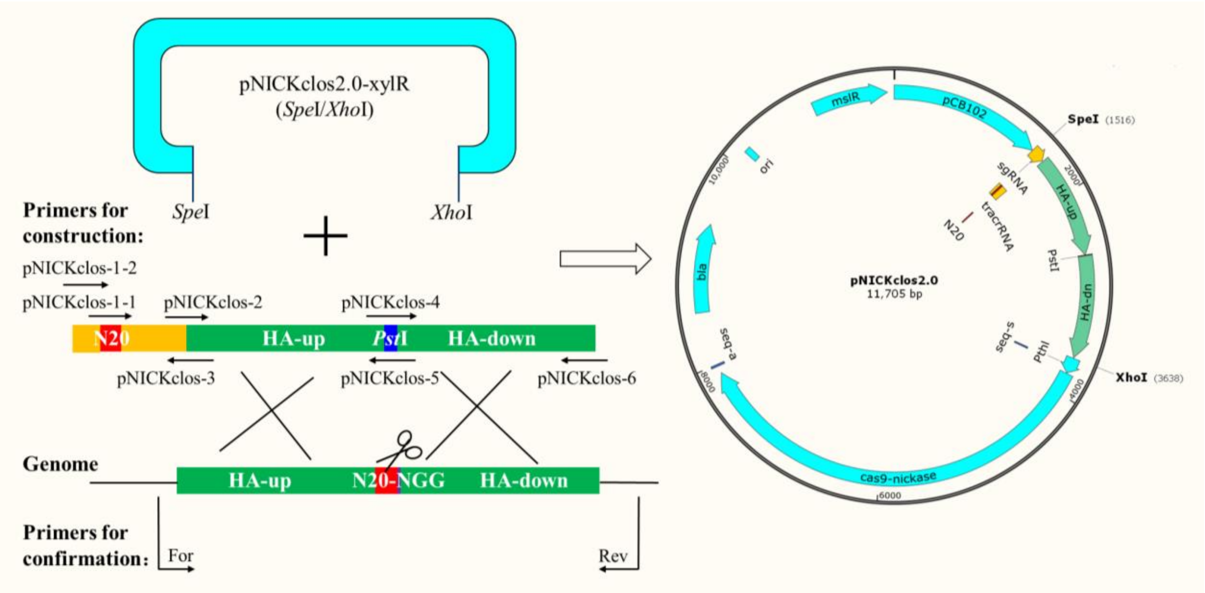

Supplementary Figure 1. Schematic diagram of the plasmid construction and gene-editing process. pNICKclos-1-2, pNICKclos-1-1, pNICKclos-2, pNICKclos-3, pNICKclos-4, pNICKclos-5, and pNICKclos-6 are primers used in the construction of pNICKclos2.0 series of plasmids. For and Rev are primers used in colony-PCR after electroporation and the PCR productions obtained are digested by restriction endonuclease and sequenced to confirm the positive transformant colonies. HA-up: Upstream homology arm. HA-down: Downstream homology arm.

References

Li, Q., Chen, J., Minton, N. P., Zhang, Y., Wen, Z., Liu, J., et al. (2016). CRISPR-based genome editing and expression control systems in *Clostridium acetobutylicum* and *Clostridium beijerinckii*. *Biotechnol. J.* 11, 961–972. doi: 10.1002/biot.201600053

Lu, C., Dong, J., and Yang, S. T. (2013). Butanol production from wood pulping hydrolysate in an integrated fermentation–gas stripping process. *Bioresour. Technol*. 143, 467–475. doi: 10.1016/j.biortech.2013.06.012
